# Functional analysis of suspected splicing variants in *CLCN5* gene in Dent disease 1

**DOI:** 10.1007/s10157-020-01876-x

**Published:** 2020-07-28

**Authors:** Tomohiko Inoue, China Nagano, Masafumi Matsuo, Tomohiko Yamamura, Nana Sakakibara, Tomoko Horinouchi, Yugo Shibagaki, Daisuke Ichikawa, Yuya Aoto, Shinya Ishiko, Shingo Ishimori, Rini Rossanti, Kazumoto Iijima, Kandai Nozu

**Affiliations:** 1 Department of Pediatrics, Kobe University Graduate School of Medicine, 7-5-1 Kusunoki-cho, Chuo-ku, Kobe, Hyogo 650-0017 Japan; 2 Division of Nephrology and Hypertension, St. Marianna University Graduate School of Medicine, 2-16-1 Sugao, Kawasaki City, Kanagawa 216-8511 Japan; 3 Department of Physical Therapy, Faculty of Rehabilitation, Kobe Gakuin University, 518 Arise, Ikawadani-cho, Nishi-ku, Kobe, Hyogo 651-2180 Japan

**Keywords:** CLCN5, Splicing, Minigene, Variant, In silico

## Abstract

**Background:**

In recent years, the elucidation of splicing abnormalities as a cause of hereditary diseases has progressed. However, there are no comprehensive reports of suspected splicing variants in the *CLCN5* gene in Dent disease cases. We reproduced gene mutations by mutagenesis, inserted the mutated genes into minigene vectors, and investigated the pathogenicity and onset mechanisms of these variants.

**Methods:**

We conducted functional splicing assays using a hybrid minigene for six suspected splicing variants (c.105G>A, c.105+5G>C, c.106−17T>G, c.393+4A>G, c.517−8A>G, c.517−3C>A) in *CLCN5*. We extracted information on these variants from the Human Gene Mutation Database. We reproduced minigene vectors with the insertion of relevant exons with suspected splicing variants. We then transfected these minigene vectors into cultured cells and extracted and analyzed the mRNA. In addition, we conducted in silico analysis to confirm our minigene assay results.

**Results:**

We successfully determined that five of these six variants are pathogenic via the production of splicing abnormalities. One showed only normal transcript production and was thus suspected of not being pathogenic (c.106−17T>G).

**Conclusion:**

We found that five *CLCN5* variants disrupted the original splice site, resulting in aberrant splicing. It is sometimes difficult to obtain mRNA from patient samples because of the fragility of mRNA or its low expression level in peripheral leukocytes. Our in vitro system can be used as an alternative to in vivo assays to determine the pathogenicity of suspected splicing variants.

**Electronic supplementary material:**

The online version of this article (10.1007/s10157-020-01876-x) contains supplementary material, which is available to authorized users.

## Introduction

Dent disease is an X-linked genetic disease of tubulopathy characterized by low-molecular-weight proteinuria, hypercalciuria, and renal calcification [1]. Approximately 60% of Dent disease cases are caused by mutations in the *CLCN5* gene, which are referred to as Dent disease 1 (OMIM#300,009). In the kidney, the *CLCN5* gene is involved in the synthesis of ClC-5 at proximal tubules, ascending limb of Henle’s loop, and the intercalated cells of the collecting duct [1]. ClC-5 is a type of voltage-gated chloride channel involved in regulating cell volume, membrane potential, and transepithelial transport. A mouse model of ClC-5 knockout demonstrated that impairment of the endocytic traffic is the major cause not only of tubular proteinuria, but also of hypercalciuria and consequently kidney stones [2].

In the Human Gene Mutation Database (HGMD) Professional (https://portal.biobase-international.com/hgmd/pro/start.php), most reported mutations of the *CLCN5* gene are missense or nonsense mutations, followed by deletions and splicing variants located at obvious splice sites within two bases immediately before and after exons (AG-GT). However, some previously reported suspected splicing variants were outside these regions [1, 3–6]. Although cases with such mutations might exhibit splicing abnormality, this has not been proven.

RNA splicing is a process involving the removal of the intervening, noncoding sequences of genes (introns) from pre-mRNA and the joining of the protein-coding sequences (exons) together, to enable translation of mRNA into a protein. Recent research has underlined the abundance and importance of splicing variants in the etiology of inherited diseases [7–11]. The most effective method to determine whether the selected variants affect splicing is to analyze the mRNA extracted from the relevant tissue of patients. However, the extraction of RNA from the affected organs is sometimes difficult. In addition, the analysis of mRNA is usually difficult because of its low expression levels in peripheral leukocytes or its fragility. In silico algorithms were developed to predict splicing abnormalities and are available as tools, but they do not always predict the exact splicing abnormalities. If the appropriate material for RNA analysis is not available, an alternative method is a minigene splicing assay—an in vitro hybrid system that allows exon trapping.

Against this background, in this study, the pathogenicity of six variants in cases of Dent disease one, in which splicing abnormalities were suspected, was analyzed using in vitro (minigene assay) and in silico assays.

## Materials and methods

Among the variants in the *CLCN5* gene reported as the causative agents of Dent disease one, we selected six variants suspected of being associated with splicing abnormalities from HGMD Professional, which were outside the obvious splice sites (not within two bases immediately before and after exons: AG-GT). In general, variants in the acceptor and donor sites (AG-GT sites) always alter the interactions between pre-mRNA and proteins involved in intron removal. We excluded these AG-GT consensus site variants from this study. Each variant is shown in Table 1.

**Table 1:** In vivo (Minigene) and in silico assays

	gDNA mutation	mRNA	In vitro (minigene assay)		In silico (MaxEnt score)			Reference
			Genetic region cloned	Result	Original score	Variant score	New splicing site score	
No. 1	c. 105 G > A	N/A	Intron 1–2	23 bp inclusion of intron 2	10.06	6.42	10.47	[1]
No. 2	c. 105 + 5 G > C	23 bp inclusion of intron 2	Intron 1–2	23 bp inclusion of intron 2	10.06	8.1	10.47	[4]
No. 3	c. 106–17 T > G	Normal	Intron 2–3	Normal	11.08	10.24		[3]
No. 4	c. 393 + 4 A > G	N/A	Intron 3–4	Exon 4 skipping	8.1	4.15		[5]
No. 5	c. 517–8 A > G	N/A	Intron 5–6	7 bp inclusion of intron 5 partial deletion (32 bp) of exon 6 partial deletion (88 bp) of exon 6 Exon 6 skipping	11.44	4.78	7.42 5.30 3.95	[1]
No. 6	c. 517–3 C > A	Exon 6 skipping	Intron 5–6	Partial deletion (32 bp) of exon 6 partial deletion (88 bp) of exon 6 Exon 6 skipping	11.44	4.78	5.30 3.95	[6]

*N/A* not available

## In vitro assay

**Fig. 1:**
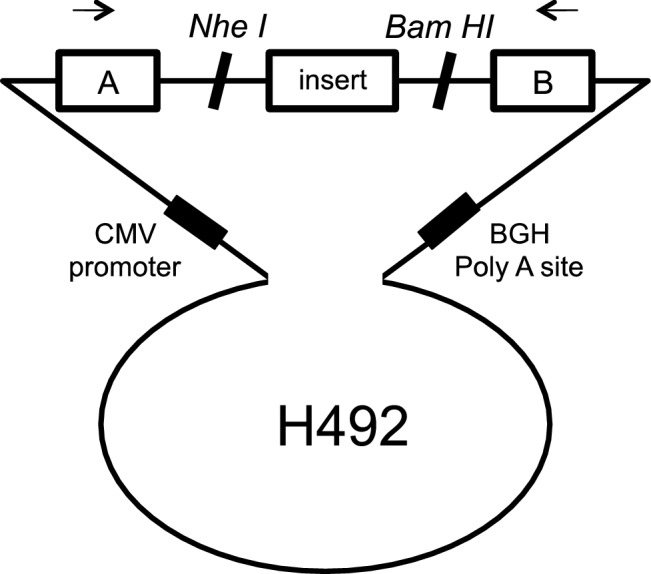
Schema for the hybrid minigene. The H492 vector contains two cassette exons, A and B, with a multiple cloning site. The H492 vector also contains a cytomegalovirus (CMV) enhancer–promotor and a bovine growth hormone gene (BGH) polyadenylation site. The arrows show the positions of the primers used in the RT-PCR assay

To create hybrid minigene constructs, we used the H492 vector based on the pcDNA 3.0 mammalian expression vector (Invitrogen, Carlsbad, CA, USA) (Fig. 1) that we developed previously [11–15]. We cloned DNA fragments containing a couple of exons and introns around the target variant in the *CLCN5* gene using In-Fusion cloning methods with In-Fusion®HD Cloning Kit (Takara Bio Inc., Kusatsu, Japan), in accordance with the manufacturer’s instructions.

Because patients’ gDNA was not available, we initiated cloning from wild-type gDNA and then introduced mutations by site-directed mutagenesis using PrimeSTAR® Mutagenesis Basal Kit (Takara Bio Inc.), in accordance with the manufacturer’s instructions. The primers used are shown in Supplementary Table 1.

The hybrid minigenes were confirmed by sequencing and transfected into HEK293T and HeLa cells using Lipofectamine® 3000 Transfection Kit (Thermo Fisher Scientific, Waltham, MA, USA). Total RNA was extracted from cells after 36 h using the RNeasy® Plus Mini Kit (QIAGEN, Hilden, Germany).

Total RNA (1 μg) was reverse-transcribed using RNA to cDNA EcoDry™ Premix (Double Primed) (Takara Bio Inc.). PCR was performed using a forward primer corresponding to a segment upstream of exon A and reverse primer complementary to a segment downstream of exon B (Fig. 1). PCR products were analyzed by electrophoresis on a 1.5% agarose gel using a 50 bp DNA ladder and direct sequencing.

## In silico assay

We predicted the splicing domain strength in each variant, using Human Splicing Finder (https://www.umd.be/HSF3/). As for potential splice sites, scores obtained using MaxEnt Scan matrix are shown in Table 1.

## Results

**Fig. 2:**
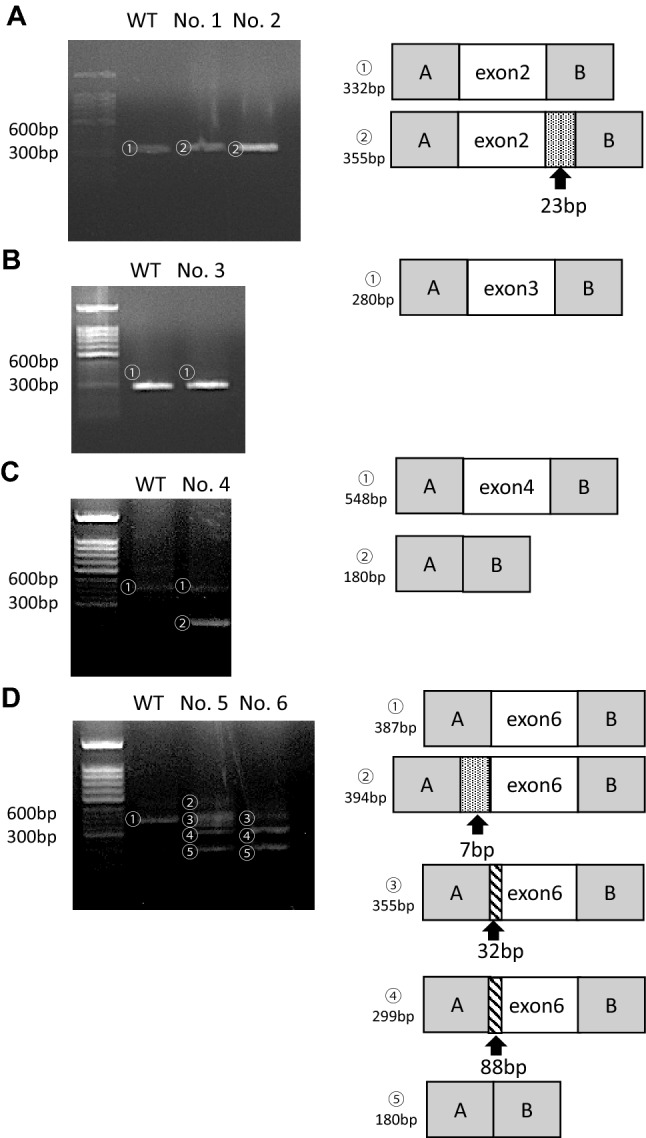
Electrophoresis results and schematic transcript analysis results from the minigene constructs. The direct sequence is shown in Supplementary Fig.1. **a** Wild type (WT) exhibited a single band (full) and No. 1 and No. 2 exhibited a single band (23 bp inclusion). **b** Wild type (WT) and No. 3 exhibited a single band (full). **c** Wild type (WT) exhibited a single band (full), while No. 4 exhibited double bands (full and exon 4 skipping). **d** Wild type (WT) exhibited a single band (full), while No. 5 exhibited quadruple bands (7 bp inclusion, 32 bp deletion, 88 bp deletion, and exon 6 skipping). No. 6 exhibited triple bands (32 bp deletion, 88 bp deletion, and exon 6 skipping)

**Fig. 3:**
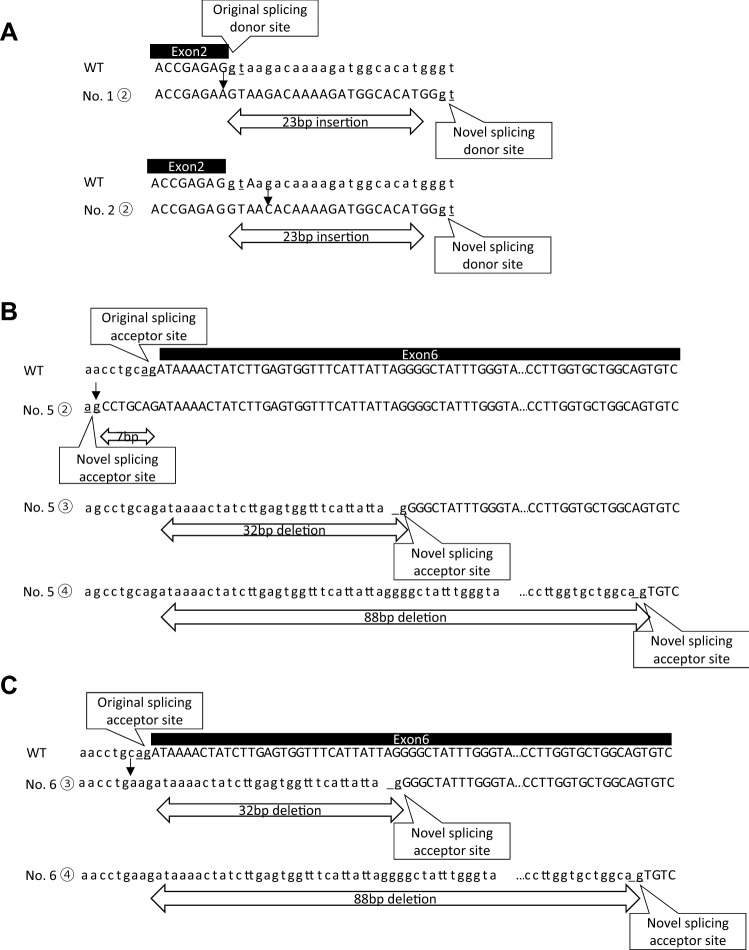
Schemas of the splicing patterns found in No. 1, No. 2, No. 5, and No. 6. **a** The splice site variants (c.105G>A and c.105+5G>C) disrupted the original donor site and resulted in the generation of an aberrant donor site that led to a 23 bp insertion. **b** The splice site variant (c.517−8A>G) disrupted the original acceptor site and resulted in the generation of an aberrant acceptor site that led to a 7 bp insertion, 32 bp deletion, and 88 bp deletion. **c** The splice site variant (c.517−3C>A) disrupted the original acceptor site and resulted in the generation of an aberrant acceptor site that led to a 32 bp deletion and an 88 bp deletion

Analysis of each cDNA revealed splicing abnormalities associated with five of the six variants (Table 1, Fig. 2, 3). We observed one transcript with a 23 bp inclusion of intron two at the end of exon two in two variants (c.105G>A and c.105+5G>C) (No. 1 and 2, Fig. 2a). In both cases, normal nucleotide sequences were not detected. We observed no change in one variant (No. 3, c.106−17T>G) (Fig. 2b). The variant c.393+4A>G led to skipping of the whole of exon 4 (No. 4, Fig. 2c). For the variant c.517−8A>G, we observed several transcripts: (1) inclusion of an intron five fragment (7 bp), (2) partial deletion (32 bp) of exon 6, (3) partial deletion (88 bp) of exon 6, and (4) exon 6 skipping (No. 5, Fig. 2d). For the variant c.517−3C>A, we observed the same transcripts as with No. 5 (No. 6, Fig. 2d), except for the inclusion of 7 bp from the intron 5 fragment (Fig. 2d).

## In silico assay

We examined the change of splicing score due to each gene variant using Human Splicing Finder. The results of the MaxEnt score are shown in Table 1. There were significant decreases in the original donor site score in No. 1 (c.105G>A), No. 2 (105+5G > C), and No. 4 (c.393+4A>G), as well as in the original acceptor site score in No. 5 (c.517−8A>G) and No. 6 (c.517−3C>A). A marked decrease in the original site score was not found for No. 3 (c.106−17T>G). These results completely matched the minigene assay results.

## Discussion

This is the first report describing a comprehensive exploration of *CLCN5* gene splicing patterns in variants reported to be pathogenic. Most of the variants showed disruption of the original splice site and the creation of new splice sites, which were shown by in vitro minigene splicing assay and confirmed by in silico analysis.

Each result can be interpreted as follows. No. 1 (c.105G>A) and No. 2 (c.105+5G>C) result in the generation of an aberrant transcript because the original donor site is disrupted, and the existing candidate new donor site in intron 2 is activated. Both of them lead to the inclusion of a 23 bp intron 2 fragment (Fig. 3a). It was also expected that No. 4 (c.393+4A>G) leads to the skipping of exon 4 due to the disruption of the original donor site of intron 4. No. 5 (c.517−8A>G) and No. 6 (c.517−3C>A) also lead to exon skipping as a result of disruption of the original acceptor site. In addition, these two variants lead to the creation of several kinds of transcript because several candidate acceptor sites are activated after the disruption of the original site. In addition, a 7 bp fragment of intron 5 is inserted because a new acceptor site is created only by No. 5 variant (c.517-8A>G) (Fig. 3b). No. 4 (c.393+4A > G), No. 5 (c.517−8A>G), and No. 6 (c.517−3C>A) lead to whole exon skipping, but No. 1 (c.105 G>A) and No. 2 (c.105+5G>C) do not. This may be due to the existence of candidate splicing sites having a large score for splicing site (Table 1, score = 10.47 in No. 1 and 2). Variant No. 3 (c.106−17T>G) was suspected of not being pathogenic, a conclusion that was derived from both in vitro and in silico splicing assays. Actually, even in the original study [3], the authors detected only normal transcript from the patient’s mRNA extracted from the peripheral leukocytes. In addition, this patient had no proteinuria but showed nephrolithiasis and hypercalciuria. This clinical information suggested that the case was unlikely to involve Dent disease.

Recently, gene targeted therapy has been developed. Splice modulation through the use of antisense oligonucleotides has emerged as an effective technique for the treatment of splicing abnormalities. The US Food and Drug Administration (FDA) approved eteplirsen (Exondys 51) as the first exon-skipping drug for the treatment of Duchenne muscular dystrophy and nusinersen (Spinraza) for spinal muscular atrophy [16]. We can also pursue the development of gene targeted therapy for cases with aberrant splicing to correct splicing patterns. The CRISPR/Cas system is also a promising tool for correcting many genetic defects [17]. For this purpose, it is critically important to determine the pathogenicity of variants of unknown significance. We believe that the results of these splicing assays are important for studying the pathogenesis and treatment of diseases.

Lourdel et al. asserted that functional studies of *CLCN5* mutations have made it possible to distinguish among the three different classes of mutation [18]. Class I mutations comprise nonsense, frameshift, and aberrant mRNA splicing mutations that lead to impairment of protein synthesis. As a consequence, Class I mutations impair processing and folding and the ClC-5 mutants of this type are retained within the endoplasmic reticulum and targeted for degradation by quality control mechanisms. We speculate that the five variants examined in this study lead to the impaired function of ClC-5.

Among three of the six variants, the original paper [3, 4, 6] showed the results of mRNA sequencing. In No. 2 (c.105+5G>C) and No. 3 (c.106−17T>G), these results were identical to our minigene assay results [3, 4]. Conversely, in No. 6 (c.517−3C>A), the mRNA sequencing results identified only exon 6 skipping [4], so this result is not completely concordant with ours. This discrepancy may be derived from the difference between in vivo and in vitro minigene assays using cultured cells. We also speculate that defense mechanisms such as nonsense-mediated decay, which eliminates abnormal mRNA in vivo, may occur in the living host [19]. Because the variants examined in this study were reported in a previous study, mRNA analysis using the patients’ samples was difficult. However, in the minigene assay, variants of interest can be inserted and their effects can be examined even when patients’ samples are not available. In addition, extracting mRNA from affected organs is often difficult. Alternatively, peripheral leukocytes are usually used for in vivo mRNA analysis; however, their mRNA expression level is usually low and the mRNA is fragile. These conditions make mRNA analysis in vivo very difficult. In such cases, the minigene assay is a good tool for assessing splicing abnormalities.

Approximately 9% of all mutations reported in the HGMD are splicing mutations (18,761/208,368) (HGMD database, accessed on October 10, 2017), although this number may well be an underestimate. Here, we assessed only variants suspected of causing splicing abnormalities. However, even variants in exons can cause splicing abnormalities. Studies for the detection of splicing abnormalities have been conducted for a long time, but it is still difficult to accurately predict abnormal splicing caused by genetic variants [20]. Recently, the number of genes identified as being responsible for inherited kidney diseases has increased rapidly. Genetic disorders are present in any part of the renal and urinary tract. Recently, using minigene assay, we have detected splicing abnormalities in the *COL4A5* gene as a cause of glomerular disease [11, 21]. In this report, we summarize splicing abnormalities associated with Dent disease 1, one of the most common renal tubulopathies. Next, we plan to analyze splicing abnormalities in the OCRL gene causing Lowe syndrome and in the GLA gene causing Fabry disease, using the same approach. Other inherited kidney diseases are also targets for this approach. Cases suspected of having inherited kidney diseases are less likely to undergo renal biopsy than other diseases. As a result, the affected organ, the kidney itself, cannot be used for splicing analysis. However, analysis using minigene assay that does not require biological samples and may provide a comprehensive understanding of splicing abnormalities in inherited kidney diseases. Further analysis is thus required to further elucidate the mechanism of splicing.

In conclusion, this study provides proof of splicing abnormalities associated with some suspected variants in the *CLCN5* gene. The incidence of genetic disorders due to splicing abnormalities has been underestimated, and further comprehensive studies should be conducted to clarify the contribution of splicing abnormalities to the onset of disease. In this study, we have focused on Dent disease, but we have also conducted a comprehensive approach for Alport syndrome [11, 21]. In future work, we should apply minigene assay to other common inherited kidney diseases such as Lowe syndrome and Fabry disease.

## Electronic supplementary material

Below is the link to the electronic supplementary material.

Supplementary file1 (DOCX 2720 kb)
