# Correction to: Functional analysis of suspected splicing variants in *CLCN5* gene in Dent disease 1

**DOI:** 10.1007/s10157-021-02041-8

**Published:** 2021-03-15

**Authors:** Tomohiko Inoue, China Nagano, Masafumi Matsuo, Tomohiko Yamamura, Nana Sakakibara, Tomoko Horinouchi, Yugo Shibagaki, Daisuke Ichikawa, Yuya Aoto, Shinya Ishiko, Shingo Ishimori, Rini Rossanti, Kazumoto Iijima, Kandai Nozu

**Affiliations:** 1 Department of Pediatrics, Kobe University Graduate School of Medicine, 7-5-1 Kusunoki-cho, Chuo-ku, Kobe, Hyogo 650-0017 Japan; 2 Division of Nephrology and Hypertension, St. Marianna University Graduate School of Medicine, 2-16-1 Sugao, Kawasaki City, Kanagawa 216-8511 Japan; 3 Department of Physical Therapy, Faculty of Rehabilitation, Kobe Gakuin University, 518 Arise, Ikawadani-cho, Nishi-ku, Kobe, Hyogo 651-2180 Japan

## Correction to: Clinical and Experimental Nephrology (2020) 24:606–612 10.1007/s10157-020-01876-x

The article “Functional analysis of suspected splicing variants in *CLCN5* gene in Dent disease 1”, written by Tomohiko Inoue · China Nagano · Masafumi Matsuo · Tomohiko Yamamura · Nana Sakakibara ·Tomoko Horinouchi · Yugo Shibagaki · Daisuke Ichikawa · Yuya Aoto · Shinya Ishiko · Shingo Ishimori ·Rini Rossanti · Kazumoto Iijima and Kandai Nozu was originally published Online First without Open Access. After publication in volume 24, issue 7, page 606–612 the author decided to opt for Open Choice and to make the article an Open Access publication. Therefore, the copyright of the article has been changed to © The Author(s) 2021 and the article is forthwith distributed under the terms of the Creative Commons Attribution 4.0 International License (https://creativecommons.org/licenses/by/4.0/), which permits use, sharing, adaptation, distribution and reproduction in any medium or format, as long as you give appropriate credit to the original author(s) and the source, provide a link to the Creative Commons licence, and indicate if changes were made. The original article has been corrected.

