# Supplementary material for: Functional analysis of suspected splicing variants in CLCN5 gene in Dent disease 1
Source: Clin Exp Nephrol. 2020 Jul 28;24(7):606–12. doi: 10.1007/s10157-020-01876-x (PMC7935734; doi:10.1007/s10157-020-01876-x)

Supplementary data

Supplementary Table 1: The primers for cloning and mutagenesis

|  | | 5’ 3’ |
| --- | --- | --- |
| Exon 2 | F | CCGTGCTTTGTTAGCTCATTTTGTGCCTACACCACA |
|  | R | TCGATGTTAACGCTAAAGCACCAACATGGCACA |
| Exon 3 | F | CCGTGCTTTGTTAGCGTCAAGAGATTGCCCTTGGA |
|  | R | TCGATGTTAACGCTACCAAAGGGCTAACATCACC |
| Exon 4 | F | CCGTGCTTTGTTAGCTAAAGACAGGCCCACTAGCC |
|  | R | TCGATGTTAACGCTAGGTTCCCCTTTCTGTGGAA |
| Exon 6 | F | CCGTGCTTTGTTAGCTATCCCTCCCTCTCCAGCTC |
|  | R | TCGATGTTAACGCTATGACAGACGCTGCACTATCC |
| No. 1 | F | ACCGAGAAGTAAGACAAAAGATGGCA |
|  | R | GTCTTACTTCTCGGTGCCTATCCCGG |
| No. 2 | F | GAGGTAACACAAAAGATGGCACATGG |
|  | R | CTTTTGTGTTACCTCTCGGTGCCTA |
| No. 3 | F | CAATGTTGTCTCATTTTCCCCTAGA |
|  | R | AATGAGACAACATTGGTACACTCTAC |
| No. 4 | F | TGAGGTAGCATGTAGTGATGTTTTA |
|  | R | CTACATGCTACCTCATCTGTGCTGA |
| No. 5 | F | GTGTTTAGCCTGCAGATAAAAACTA |
|  | R | CTGCAGGCTAAACACAGAAAGATTGA |
| No. 6 | F | TAACCTGAAGATAAAAACTATCTTGA |
|  | R | TTTATCTTCAGGTTAAACACAGAAAG |
| H492  plasmid | F | ATTACTCGCTCAGAAGCTGTGTTGC |
|  | R | AAGTCTCTCACTTAGCAACTGGCAG |

Supplementary Figure 1.

**A**① WT full


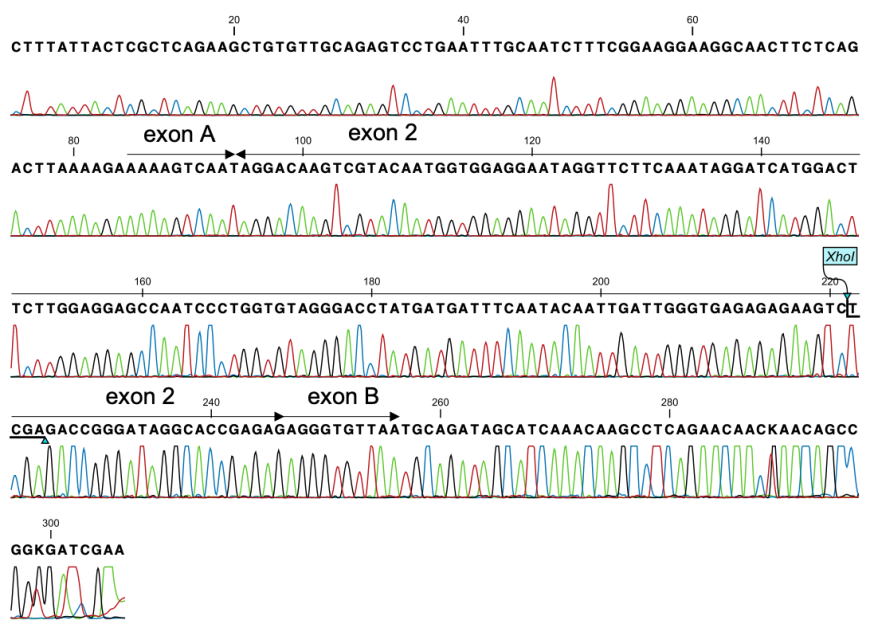


A②　No. 1/No. 2 23 bp insertion


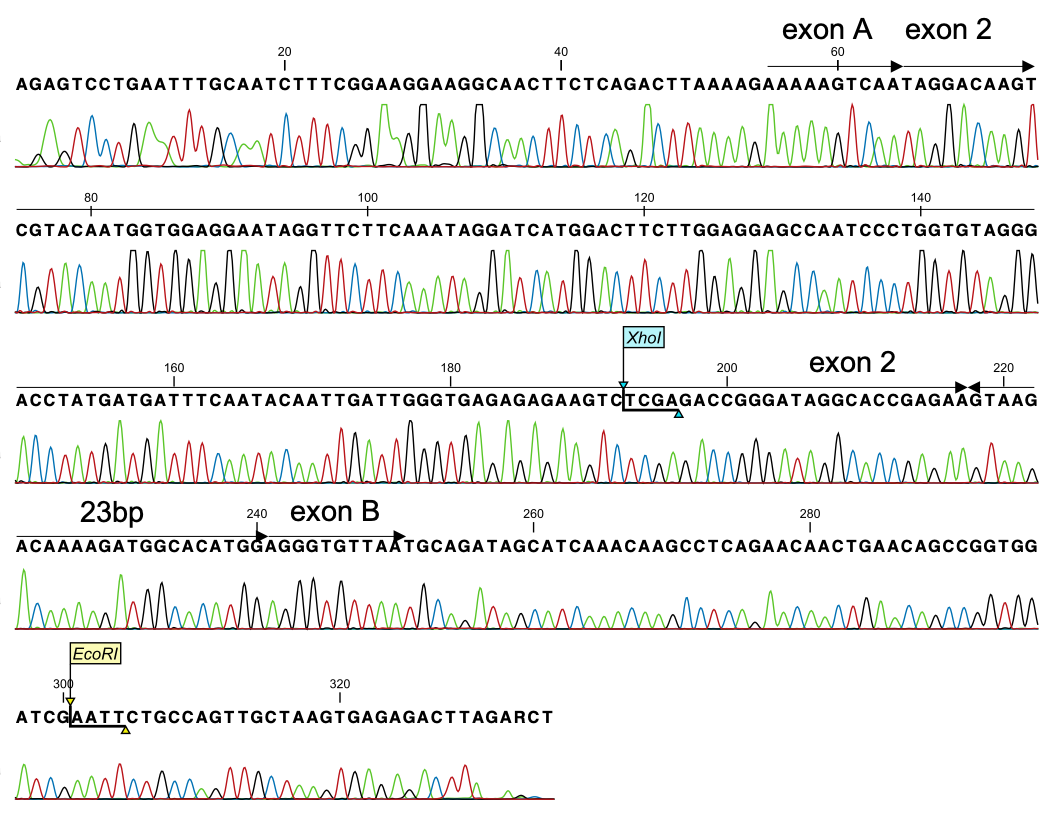


B① WT/ No. 3 full


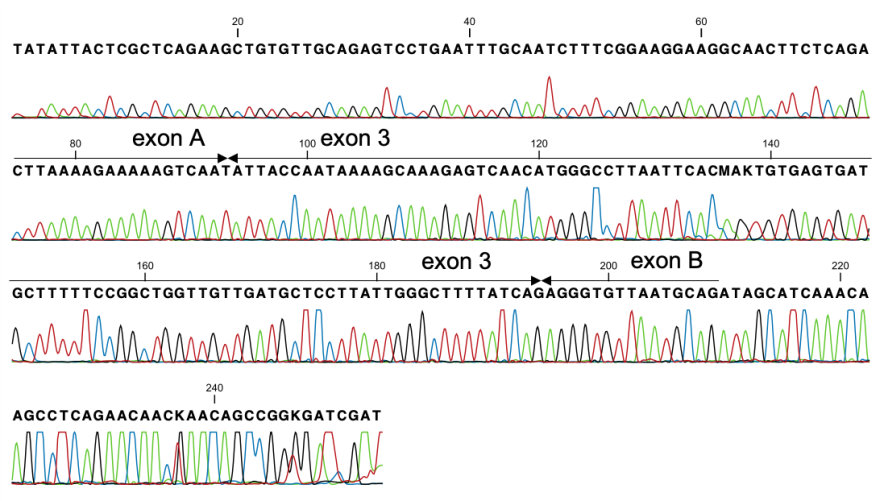


C① WT/No. 4 full


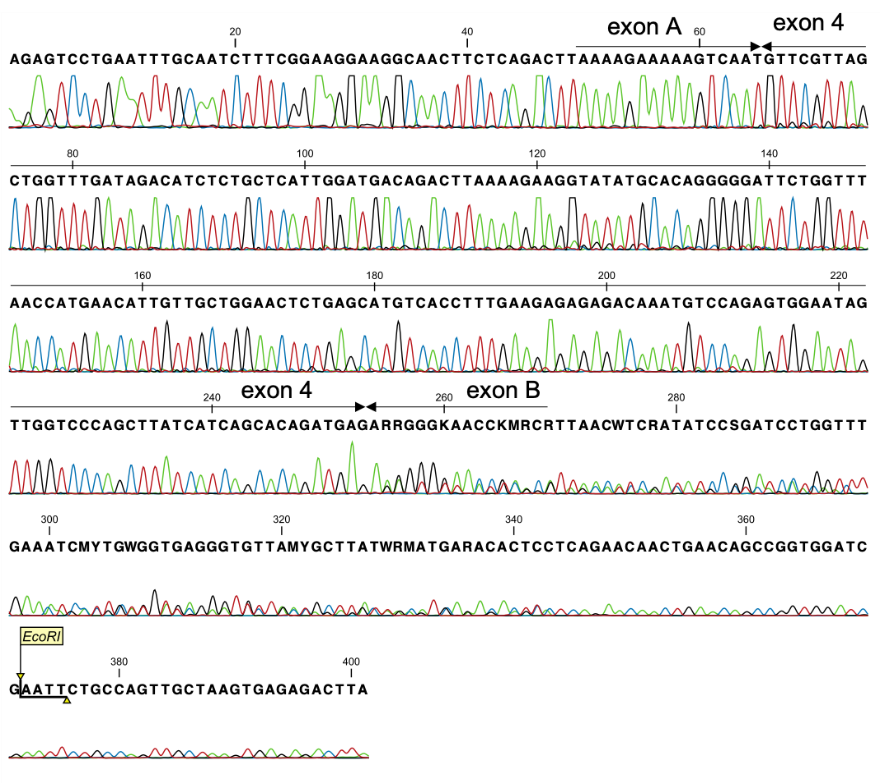


C② No. 4 Exon 4 skipping


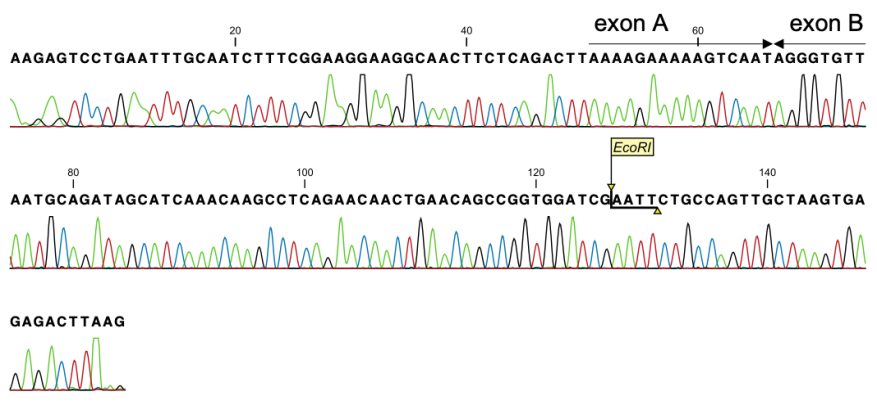


D① WT full


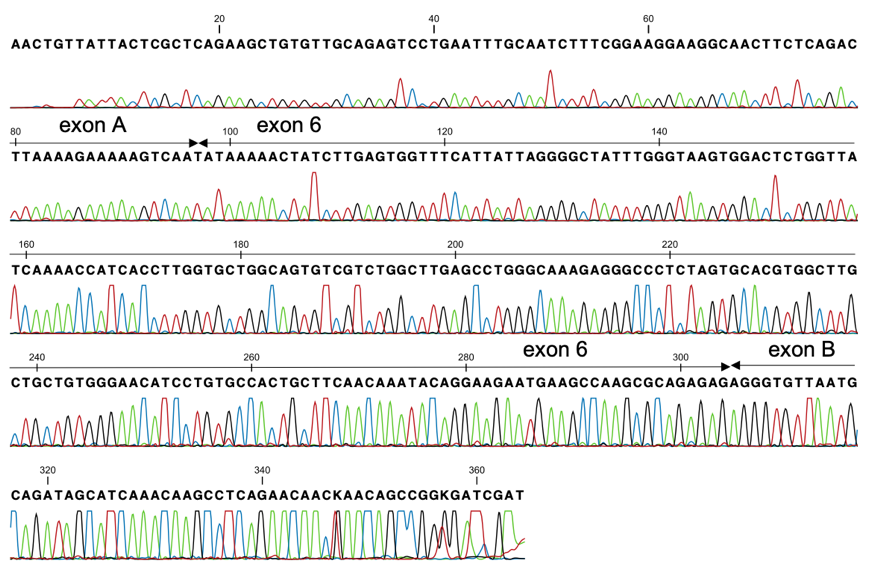


D② No. 5 7 bp insertion


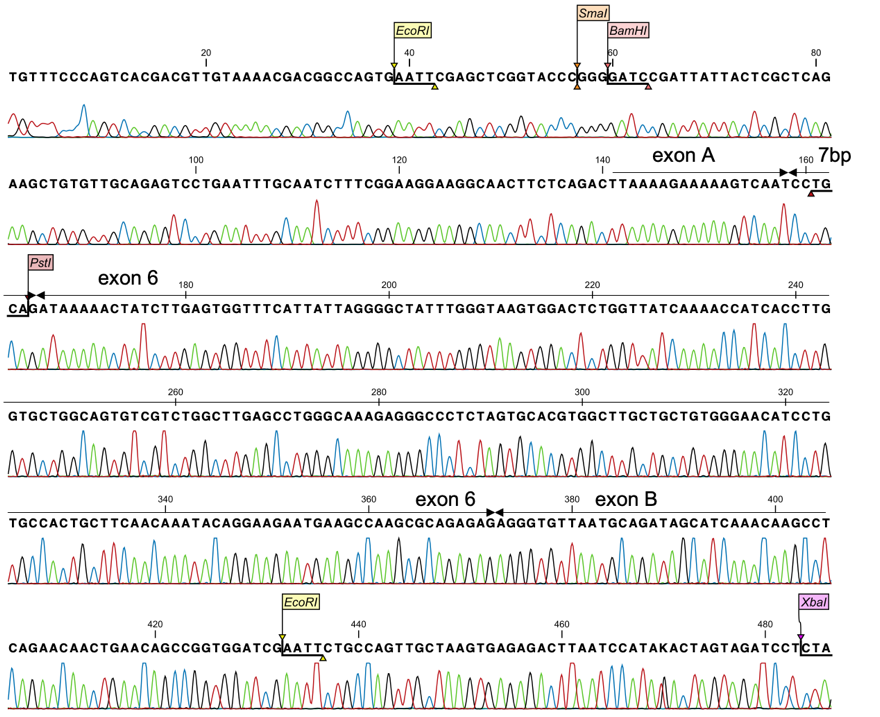


D③ No. 5/No. 6 32 bp deletion


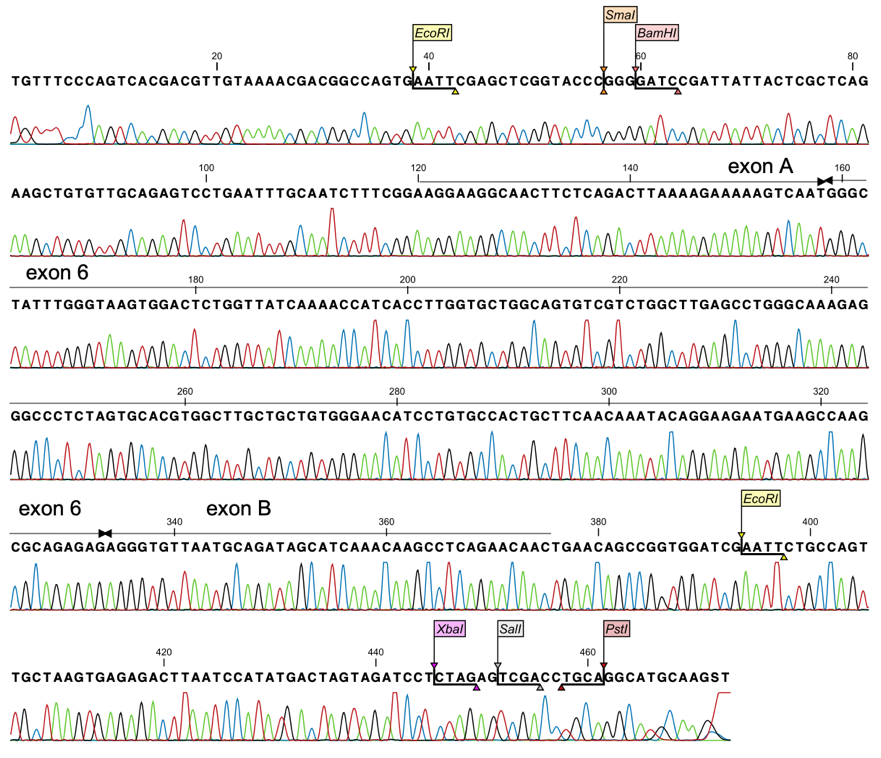


D④ No. 5/No. 6 88 bp deletion


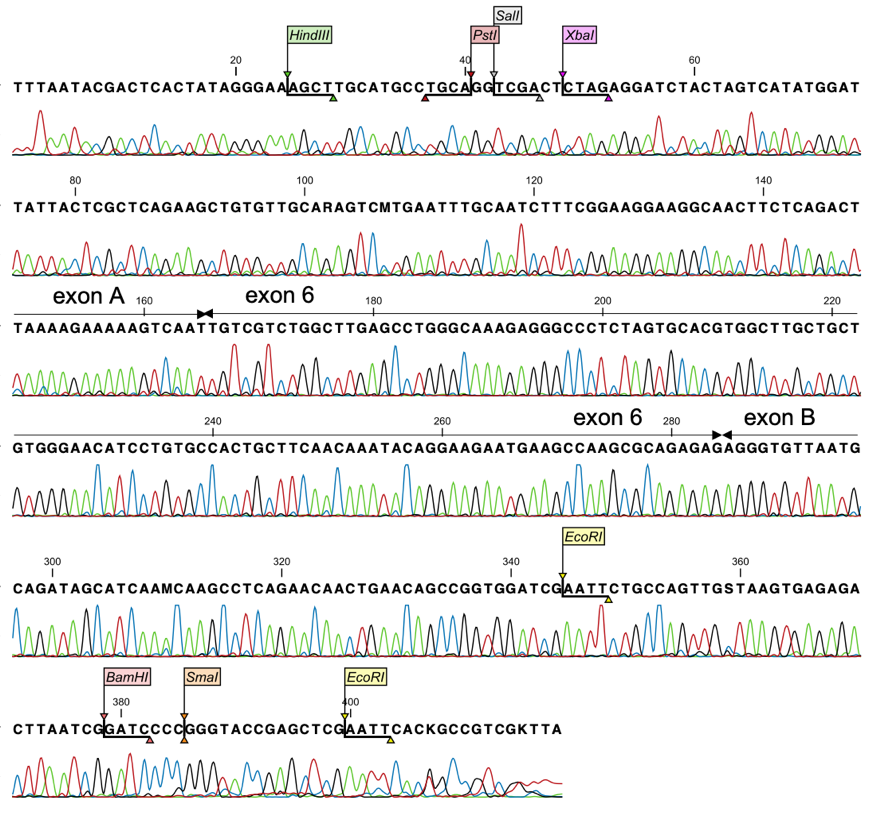


D⑤ No. 5/No. 6 Exon 6 skipping


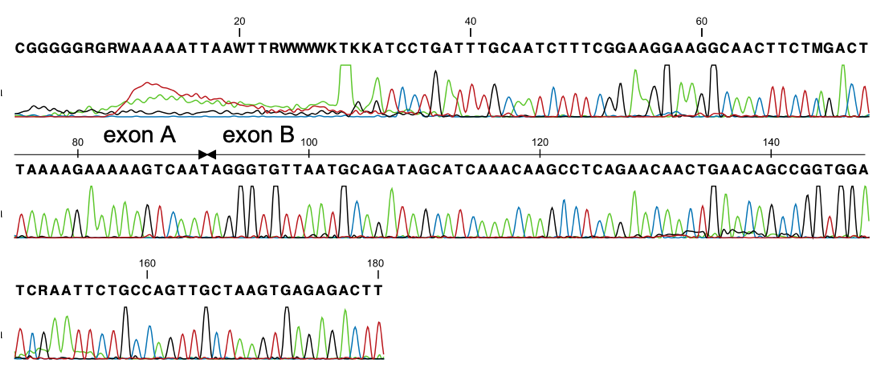

Supplement: Supplementary file 1 — Supplementary file1 (DOCX 2720 kb) [file 10157_2020_1876_MOESM1_ESM.docx]
